# Clinical Longevity of Indirect Composite Resin Inlays and Onlays: An Up to 9-Year Prospective Study

**DOI:** 10.1055/s-0041-1735420

**Published:** 2021-11-08

**Authors:** Aristidis Galiatsatos, Panagiotis Galiatsatos, Dimitra Bergou

**Affiliations:** 1 Division of Dental Technology, Department of Biomedical Sciences, University of West Attica, Athens, Greece; 2 School of Dentistry, National and Kapodistrian University of Athens, Athens, Greece; 3 Private Practice, Athens, Greece

**Keywords:** adhesion, clinical longevity, esthetics, indirect composite, inlay, marginal discoloration, onlay, restorative dentistry

## Abstract

**Objective** This clinical study evaluated the clinical performance of composite resin inlays and onlays over 9 years.

**Materials and Methods** Sixty composite resin inlays and onlays were placed in 32 patients, aged 20 to 60 years, by a single operator using the same clinical procedure. The restorations were examined for fracture rate; esthetics; and patient acceptance and marginal integrity, including caries, marginal discoloration, tooth integrity, and surface texture. All restorations were evaluated at the time of placement and 3, 6, and 9 years after placement by using the modified U.S. Public Health Service criteria.

**Results** At the 3-year follow-up, an Alpha score was given to 88.4% of restorations, while a Bravo score was given to the remaining 11.6%. There was not any failure. At the 6-year follow-up, the success rate of the restorations was 100% without failure. None of the restorations was scored with Delta (D). An Alpha score was given to 60% of the restorations, a Bravo score was assigned to 35%, and a Charlie score was 5% of the restorations. Overall, the success rate of the restorations at 9-year follow-up was 85% and the failure rate was 15%. An Alpha score was given to 15% of the restorations, a Bravo score was given to 50%, a Charlie score was assigned to 20%, and a D score was given to 15% of the restorations.

**Conclusion** Indirect resin composite inlays and onlays showed acceptable long-term clinical results. The success rate of the restorations at 9-year follow-up was 85% and the failure rate was 15%.

## Introduction

The quest for an esthetic posterior restoration that is both conservative and predictable has plagued the dental profession for many years. Under this theory, indirect inlays and onlays have been used increasingly in the last decades.

In today’s dental practice, there are many materials and solutions available to restore a partially damaged posterior tooth. The development of reinforcing ceramic systems, coupled with the ability to etch and bond the porcelain to the underlying etched tooth structure, has allowed these types of restoration to become a part of today’s operative armamentarium. Many clinical studies examined the performance of ceramic inlays and onlays for varying serving times with very good results. Unfortunately, ceramics have some disadvantages, such as low tensile strength, excessive brittleness, fracture, and time-consuming laboratory procedures. 1 2 3 4

In response to the limitations of ceramics, new polymer-based resin-composite materials have been developed. This new category of materials referred to as hybrid polymers or hybrid ceramics, also known as resin-matrix-ceramics, resin-based ceramics, or nanoceramics represents the synergy of ceramics and composites with their respective beneficial mechanical properties. Some of them are normally stress distribution; reduced polymerization shrinkage; very good wear resistance; and excellent characterization and adjustment of the occlusal surface, reparability, and easy manipulation compared with ceramic. 5 6 7 8 9 10 11 12 13 Furthermore, it appears that these materials meet the increasing demands of the patients for an esthetic appearance of the posterior teeth. Today, there are many new polymeric restorative materials for indirect applications ( Table 1 ). 8 9 10 11

**Table 1:** Modern polymeric systems for indirect restorations

Material	Manufacturer
Artglass	Heraeus/Kulzer
Signum	Heraeus/Kulzer
Sinfony	3M - ESPE
Ceramage	Shofu
Solidex	Shofu
Gradia Indirect, Plus	GC Corp
Targis	Ivoclar–Vivadent
SR Adoro	Ivoclar–Vivadent
SR Nexco	Ivoclar–Vivadent
Belleglass	Kerr
Estenia	Kuraray
Premise indirect	Kerr
Sculpture	Jeneric/Pentron

Searching the bibliographical sources, the number of long-term clinical studies regarding the performance of these materials is limited. So, the purpose of this clinical study was to evaluate the clinical performance of composite inlays and onlays longitudinally over 9 years.

## Materials and Methods

Τhis clinical survey involved 32 patients, the age and gender of which are shown in Table 2 . The 32 patients required aesthetic and functional treatment in their posterior teeth due to dental caries, recurrent caries, or replacement of old amalgam fillings. They received 60 inlays and onlays in a private dental office in Athens. All of the treated teeth were vital. All patients accepted their participation in the research protocol and agreed to a recall program for 9 years, consisting of one appointment every 3 years. This survey excluded patients with a high index of caries, poor level of oral hygiene, malfunctions (impacted and/or misaligned teeth, malocclusion), active periodontal or/and pulpal disease, and parafunctional habits like bruxism. None of the patients dropped out or were dismissed. Table 3 shows the distribution of the teeth receiving indirect restorations. The restorations included 18 one-surface inlays, 20 two-surfaces inlays, 10 three-surfaces inlays, and 12 onlays.

**Table 2:** Age and gender of patients

Age (y)	Men	Women	Total
20–30	3	8	11
31–40	5	5	10
41–50	3	3	6
51–60	3	2	5
Total	14	18	32

**Table 3:** Distribution of the teeth receiving indirect restorations

Tooth	Maxilla	Mandible	Total
First premolar	5	12	17
Second premolar	8	10	18
First molar	8	9	17
Second molar	3	5	8
Total	24	36	60

All clinical procedures were performed by one clinician, and all materials were used according to the recommendations of the manufactures.

For the tooth preparations, the basic principles for adhesive restorations were followed.

The cavity form was developed as conservatively as possible; only the compromised portion of the tooth was removed and convenient access was provided for the subsequent restoration. The axial walls of the cavity preparation were prepared with a 6- to 10-degree taper by using appropriate diamond burs (Inlay Prep-set, Intensive, Viganello-Lugano, Switzerland), which allows easier placement and removal of the restoration during the try-in phase. For the onlay preparation, a 1.5- to 2.0-mm reduction in vertical height of the cusp and all occluding areas and rounded occlusal-axial angles, and hollow–ground chamfer finish line was necessary. Special diamond burs were used for tooth preparation. (Inlay Prep-set, Intensive, Viganello-Lugano, Switzerland). Where necessary, a glass-ionomer base in thickness greater than 1.5 mm was placed on the pulpal floor for protection.

The final impression was made with a siloxane material (Speedex, Coltene -Whaledent Co.). An impression of the opposite teeth was made, using an alginate impression material (Cavex impressional, Cavex Co.). Also, an interocclusal record was made (Luxa Bite, DMG Co.). After impression making, the teeth were provisionalized with a light-cured provisional material (Clip Light-Cured Provisional Filling, Voco Co.).

One dental technician made all the restorations using a modern polymeric system (Gradia, GC) according to the manufacturer's instructions.

The bonding procedure for each tooth was conducted under a rubber dam isolation. The cavity preparation was cleaned with a wet slurry of flour pumice and then was treated with a 37% phosphoric acid gel etchant (Etching gel, DMP Co, Greece). After etching and drying, the dentin adhesive system (Gluma 2 Bond, Kulzer Co, Germany) was applied uniformly and gently air thinned. The restoration was cleaned in the ultrasonic cleaner and then washed and dried. After that, it was coated with a silane coupling agent (Monobond S, Ivoclar, Vivadent). Finally, the restoration was luted adhesively with composite resin cement (Variolink II, Ivoclar, Vivadent). The super floss beneath the contact point was drawn through to remove the excess resin interproximally. The restoration was held firmly in place and was light-cured from all aspects—proximal, facial, lingual, and occlusal—for 60 seconds each. Once the restoration bonded into position and cured completely, the final occlusal adjustment was made. Finally, the finishing procedures were done with a series of microfine diamond strips, multifluted finishing burs, and polishing disks specifically designed for the finishing process (Ιntensive metal diamond strips, Intensive proxostrip, Composhape set A&P Intensive, Viganello-Lugano, Switzerland).

Patients were given detailed oral hygiene instructions and asked to inform the clinician of any problem that occurs in the treated teeth.

## Evaluation

Each restoration was evaluated at baseline and 3, 6, and 9 years, with mirror, probe, and a dental loupe (×4.5), by another clinician who was not involved in the clinical procedures. The evaluation followed the modified U.S. Public Health Service (USPHS) criteria for the following parameters: surface texture, color match, marginal adaptation, marginal discoloration, restoration integrity (fracture), tooth integrity, sensitivity, and patient satisfaction ( Tables 4 and 5 ). 4 14 15 16 Descriptive analysis was performed for the evaluation of the restorations and the tooth outcome according to the modified USPHS criteria.

**Table 4:** Modified U.S. Public Health Service criteria for evaluation

Modified USPHS criteria	Description	Score
Excellent/good	Perfect without fault, or slight deviations from ideal performance, correction possible without damage of tooth or restoration	Alpha
Acceptable	Small defects, every clinical intervention is performed without damaging the tooth or the restoration and no negative effect is expected	Bravo
Unacceptable but repairable	Serious defects, the restoration/tooth needs to be repaired	Charlie
Poor/failure	Immediate replacement necessary	Delta
Abbreviation: USPHS, U.S. Public Health Service.		

**Table 5:** Descriptive criteria used for scoring restoration quality

Parameter	Alpha (A)	Bravo (B)	Charlie (C)	Delta (D)
Surface texture	Completely smooth surface	Slightly rough surface or with small notches, loss of gloss	Surface with visual and tactile roughness with visual cracks and notches	Visibly damaged surface, pits, and grooves throughout the material
Color match	Corresponding color between the tooth and the restoration	Moderate mismatch in color, shade, or translucency	Extensive color mismatch, outside the limits of acceptable appearance	Gross mismatch
Marginal adaptation	No cracks/gaps are visible along the margins of the restoration, the probe does not catch	The probe slightly catches along the margins	Visible cracks/gaps or extensive probe penetration between cavity wall and restoration	The restoration is either fractured, missing, or movable
Marginal discoloration	No discoloration or minor staining can be polished	Moderate surface staining, not esthetically unacceptable	Surface staining present on the restoration, intervention necessary	Severe staining and/or subsurface staining
Restoration integrity	No defects in material, no cracks, or fractures	Two or more cracks and/or chipping, but not affecting the marginal integrity or proximal contact	Chipping fractures that affect the marginal quality or proximal contact	Partial or complete loss of the restoration
Tooth integrity	No enamel defects/chipping	Visible enamel cracking, no exposed dentin	Major enamel cracking with dentin or base exposed, probe penetrates	Cusp or tooth fracture
Sensitivity	A normal reaction to cold spray compared with nonrestored teeth	Cold sensitivity has increased	Spontaneous pain referred by the patient	The tooth does not show signs of vitality
Patient satisfaction	Satisfied	Complained about the esthetic outcome	Requested an improvement	Completely dissatisfied

## Results

A total of 60 inlays and onlays were bonded on posterior teeth in 32 patients. All patients underwent the recall program. Thus, no dropout was experienced at 9 years (100%).

The results for the evaluated factors at the follow-up periods are given in Table 6 .

**Table 6:** Frequency distribution of the scores for the evaluated criteria at the follow-up periods

Category/rating	Baseline *n* , %	3 y *n* , %	6 y *n* , %	9 y *n* , %
**Surface texture**				
A	60 (100.0)	58 (96.66)	55 (91.66)	50 (83.33)
B	0 (0.0)	2 (3.33)	4 (6.66)	6 (10.00)
C	0 (0.0)	0 (0.0)	1 (1.66)	3 (5.00)
D	0 (0.0)	0 (0.0)	0 (0.0)	1 (1.66)
Color match				
A	60 (100.00)	59 (98.33)	56 (93.33)	53 (88.33)
B	0 (0.0)	1 (1.66)	4 (6.66)	6 (10.00)
C	0 (0.0)	0 (0.0)	0 (0.0)	1 (1.66)
D	0 (0.0)	0 (0.0)	0 (0.0)	0 (0.0)
Marginal adaptation				
A	60 (100.00)	58 (96.66)	54 (90.00)	47 (78.33)
B	0 (0.0)	2 (3.33)	5 (8.33)	7 (11.66)
C	0 (0.0)	0 (0.0)	1 (1.66)	3 (5.00)
D	0 (0.0)	0 (0.0)	0 (0.0)	3 (5.00)
Marginal discoloration				
A	60 (100.00)	58 (96.66)	54 (90.00)	47 (78.33)
B	0 (0.0)	2 (3.33)	5 (8.33)	7 (11.66)
C	0 (0.0)	0 (0.0)	1 (1.66)	3 (5.00)
D	0 (0.0)	0 (0.0)	0 (0.0)	3 (5.00)
Restoration integrity				
A	60 (100.00)	60 (100.00)	59 (98.33)	57 (95.00)
B	0 (0.0)	0 (0.0)	1 (1.66)	2 (3.33)
C	0 (0.0)	0 (0.0)	0 (0.0)	1 (1.66)
D	0 (0.0)	0 (0.0)	0 (0.0)	0 (0.0)
Tooth integrity				
A	60 (100.00)	60 (100.00)	58 (96.66)	55 (91.66)
B	0 (0.0)	0 (0.0)	2 (3.33)	2 (3.33)
C	0 (0.0)	0 (0.0)	0 (0.0)	1 (1.66)
D	0 (0.0)	0 (0.0)	0 (0.0)	2 (3.33)
Sensitivity				
A	55 (91.66)	60 (100.00)	60 (100.00)	56 (93.33)
B	5 (8.33)	0 (0.0)	0 (0.0)	3 (5.00)
C	0 (0.0)	0 (0.0)	0 (0.0)	1 (1.66)
D	0 (0.0)	0 (0.0)	0 (0.0)	0 (0.0)
Patient satisfaction				
A	60 (100.00)	60 (100.00)	56 (93.33)	49 (81.66)
B	0 (0.0)	0 (0.0)	3 (5.00)	7 (11.66)
C	0 (0.0)	0 (0.0)	1 (1.66)	3 (5.00)
D	0 (0.0)	0 (0.0)	0 (0.0)	1 (1.66)
Abbreviations: A, Alpha; B, Bravo; C, Charlie; D: Delta.				

At the baseline and the 3-year follow-up, all restorations obtained an Alpha score for the criteria: “restoration integrity” and “tooth integrity.”

At the 3-year examination, an Alpha score was given to 88.4% of restorations, while a Bravo score was given to the remaining 11.6%. There was not any failure.

At the 6-year follow-up, the success rate of the restorations was 100% without failure. None of the restorations was scored with Delta (D). An Alpha score was given to 60% of the restorations, a Bravo score was assigned to 35%, and a Charlie score was 5% of the restorations.

Overall, the success rate of the restorations at 9-year follow-up was 85% and the failure rate was 15%. An Alpha score was given to 15% of the restorations, a Bravo score was given to 50%, a Charlie score was assigned to 20%, and a D score was given to 15% of the restorations. At 9-year examination with D scored: one restoration for the “surface texture,” two restorations for the “tooth integrity” (small cusp fracture), and three restorations for “marginal adaptation” and also three restorations for “marginal discoloration.”

Of the 60 teeth restored, five of them showed sensitivity in the first week immediately after bonding. This slight sensitivity was resolved by the second week and completely remitted without long-term consequences. There was no additional sensitivity reported in any of the restorations through the 3- and 6-year recall. At a 9-year recall, three teeth (molars) had increased cold sensitivity (scored B) and one (premolar) had spontaneous pain referred by the patient (scored C).

Regarding the criterion “patient satisfaction,” at the 9-year follow-up, the Alpha score was 81.66%. There was only one patient completely dissatisfied ( Table 6 ).

## Discussion

This study was evaluated the clinical performance of indirect polymer composite (Gradia) inlays and onlays in posterior teeth by using modified USPHS criteria.

In the 1970s, Cvar and Ryge 17 introduced an intraoral evaluation system, known as the USPHS method, which was used to clinically evaluate resin composite restorations in posterior teeth. Over the years, this method has received many improvements so that it is reliable and valid. Today, many clinical trials use the USPHS criteria to evaluate posterior restorations. 4 18 19 20 21

Ceramic or composite inlays and onlays, also known as “esthetic inlays/onlays,” have become viable solutions for partially damaged posterior teeth. These restorations offer many advantages over comparable restorations: they restore strength to compromised teeth, they are more esthetic, and they are highly conservative.

The choice between ceramic or composite as restorative materials has become increasingly complicated since composite materials have improved in their physicomechanical properties, wear resistance, and esthetic potential.

So, this clinical study evaluated the clinical performance of composite resin inlays and onlays made of an improved polymeric material (Gradia, GC) over 9 years.

This polymeric material (Gradia, GC) contains microfine ceramic pre-polymer fillers with urethane dimethacrylate matrix. From many studies, it has been found that this material has very good physicomechanical properties, such as high strength, wear-resistance, and superior polishability for such restorations. 10 22 23

In this clinical study, at the 3-year and 6-year follow-up, the success rate of the restorations was 100% without failure. At a 9-year follow-up, the success rate was 85% and the failure rate was 15%.

It is important to distinguish between early failures (at baseline or after a few weeks/ months), from a medium time frame (3–6 years), and late failures (6–9 years). Up to 6 years, the color match, restoration integrity, tooth integrity, and sensitivity were acceptable and did not show a significant difference. None of the restorations was scored with D. An Alpha score was given to 60% of the restorations, a Bravo score was assigned to 35%, and a Charlie score was 5% of the restorations.

On the other hand, late failures occurred at 9-year follow-up with a total failure rate of 15%. Especially, nine restorations were graded with D, and only one patient was unsatisfied. The main reasons for failures were tooth integrity, surface texture, and marginal discoloration/adaptation.

About tooth integrity, there were only two teeth (one molar and one premolar) that occurred small cusp fracture at 9 years. The teeth had no clinical symptoms, as the restorations remained intact and boned in place and the fractures repaired with composite resin. Although the failure rate was very small, it should be concluded that preparation design has a significant effect on the risk of tooth fracture. Tooth preparation should follow the basic principles for these restorations. Some of them are as follows: (1) enamel should be supported by sound, healthy dentine, (2) well-rounded angles on the cuspal preparation, to prevent the propagation of restoration fracture from these sharp stress point, (3) all axial walls should be prepared with a 6- to 10-degree taper, which allows easier placement and removal of the restoration during the try-in phase, and (4) a 1.5- to 2.0-mm reduction in vertical height of the cups and all occluding areas.

About the surface texture, there was only one restoration scored with D at the end of the 9-year follow-up. This finding is most likely due to the improved mechanical properties of modern composite materials. The process of laboratory polymerization facilitates the improvement of conversion of reactable C=C double bonds and a reduction of residual internal stresses, yielding better mechanical properties. 18 24

Marginal discoloration and marginal disintegration were detected in three cases respectively at the end of the 9-year follow-up. It was hypothesized that these phenomena were interrelated and that both would deteriorate with time. These results could be related to the resin composite luting cement, which considered as the weakest point for these kinds of restorations. 11 18 19 22 25 Because adhesive inlays and onlays are inserted into the cavities with e resin cement, the luting gap is always susceptible to increased wear, as the mechanical properties of the cement are inferior, compared with the highly wear-resistant, postcured polymeric restorations. Loss of marginal adaptation is often due to polymerization shrinkage or removal of cement with instruments from the margins. The extent of microleakage depends on the extension of the margins on enamel or dentin. 26 This happens because even though polymerization shrinkage is the same, regardless of the location of margins, microleakage is greater when the cementation is made on dentine. This problem is supposedly related to dentin dehydration and the compression of the frail collagen network exposed by dentin etching during restoration insertion, while the hybrid layer structure is not stabilized by cured bonding resin. Besides, the cement at the margins is exposed to corrosion or degradation due to the activity of enzymes, changes in pH that occur always in the mouth, and also due to mechanical detachment. Also, the type of resin cement (light-cured or dual-cured) combined with the time of polymerization may affect this microleakage. All these factors were responsible for the development of marginal gaps. The pigmentation molecules are apart to stay and absorb at disintegrated margins caused by microfracture of wear of resin cement. These disintegrated areas remain and will expand with time. 4 27 Thus, this marginal disintegration will continue to exist or to increase along with the marginal discoloration. Discoloration of the margins also can be attributed to staining with pigments from food, coffee, and beverages, as well as smoking. Generally, the wear resistance of luting cement has been regarded with skepticism, regardless of the inlay/onlay material. 4 19 28 29 30 31 32

Several clinical studies on indirect composite resins inlays and onlays reported no failure or low failure rates. 11 18 19 21 22 In a 12-year study of clinical performance of one-two and multisurface composite resin inlays on premolars and molars, the failure rate was only 12%. 19 In another study, after 3 years of clinical service, 93% of the composite inlays in posterior teeth showed satisfactory results. 18 Dukic et al 21 reached a 70.7% success rate 36 months after placement and concluded that indirect composite resin restorations represent a good choice for the therapy of severely damaged teeth. Leirskar quotes 95% clinically successful results for three kinds of indirect resin composite inlays/onlays after 4 to 6 years. 33 Thordrup 34 shows that after 10 years around 80% of the inlays placed were in function. Tunac et al 35 evaluated the 2-year clinical performance of computer-aided design/computer-aided manufacturing resin composite inlay restorations in comparison with direct resin composite restorations. They concluded that all restorations were ideal or clinically acceptable in class II cavities.

## Conclusion

This clinical survey was evaluated the clinical performance of 60 indirect polymer composite (Gradia) inlays and onlays placed in 32 patients for 9 years by using modified USPHS criteria. The success rate of the restorations at 9-year follow-up was 85% and the failure rate was 15%. An Alpha score was given to 15% of the restorations, a Bravo score was given to 50%, a Charlie score was assigned to 20%, and a D score was given to 15% of the restorations. In conclusion, this kind of restoration seems clinically acceptable as a conservative and esthetic method for molar and premolar restoration.
